# Excitation energy-dependent nature of Raman scattering spectrum in GaInNAs/GaAs quantum well structures

**DOI:** 10.1186/1556-276X-7-656

**Published:** 2012-11-28

**Authors:** Ayse Erol, Elif Akalin, Fahrettin Sarcan, Omer Donmez, Sevim Akyuz, Cetin M Arikan, Janne Puustinen, Mircea Guina

**Affiliations:** 1 Physics Department, Science Faculty, Istanbul University, Istanbul, 34134, Turkey; 2 Physics Department, Faculty of Science and Letters, Istanbul Kultur University, Istanbul, 34156, Turkey; 3 Optoelectronics Research Centre, Tampere University of Technology, Korkeakoulunkatu, Tampere, 33720, Finland

**Keywords:** GaInNAs, Photoluminescence, Raman, FT-Raman, FT-IR, Local modes, 71.55.Eq; 63.22.+m

## Abstract

The excitation energy-dependent nature of Raman scattering spectrum, vibration, electronic or both, has been studied using different excitation sources on as-grown and annealed n- and p-type modulation-doped Ga_1 − *x*_In_*x*_N_*y*_As_1 − *y*_/GaAs quantum well structures. The samples were grown by molecular beam technique with different N concentrations (*y* = 0%, 0.9%, 1.2%, 1.7%) at the same In concentration of 32%. Micro-Raman measurements have been carried out using 532 and 758 nm lines of diode lasers, and the 1064 nm line of the Nd-YAG laser has been used for Fourier transform-Raman scattering measurements. Raman scattering measurements with different excitation sources have revealed that the excitation energy is the decisive mechanism on the nature of the Raman scattering spectrum. When the excitation energy is close to the electronic band gap energy of any constituent semiconductor materials in the sample, electronic transition dominates the spectrum, leading to a very broad peak. In the condition that the excitation energy is much higher than the band gap energy, only vibrational modes contribute to the Raman scattering spectrum of the samples. Line shapes of the Raman scattering spectrum with the 785 and 1064 nm lines of lasers have been observed to be very broad peaks, whose absolute peak energy values are in good agreement with the ones obtained from photoluminescence measurements. On the other hand, Raman scattering spectrum with the 532 nm line has exhibited only vibrational modes. As a complementary tool of Raman scattering measurements with the excitation source of 532 nm, which shows weak vibrational transitions, attenuated total reflectance infrared spectroscopy has been also carried out. The results exhibited that the nature of the Raman scattering spectrum is strongly excitation energy-dependent, and with suitable excitation energy, electronic and/or vibrational transitions can be investigated.

## Background

Raman spectroscopy has been a useful instrumental tool for characterization of semiconductors in terms of crystal quality, strain-induced effects, impurity modes and phonon energies [1,2]. Recently, it has been shown that Raman spectroscopy is also a powerful method to determine carrier effective mass and carrier concentration of doped semiconductors by analysing the line shape of LO-plasmon coupled modes [3]. Raman scattering is based on the inelastic scattering process of monochromatic light and mainly gives valuable information about vibrational normal modes. The energy of excitation source in conventional Raman spectroscopy is much higher than the phonon energies; therefore, the process does not only involve electron–phonon interaction, but also creates electron–hole pairs via absorption and then annihilation of electron–hole pairs via recombination. The radiative recombination process originating from transitions between electronic states can dominate the spectra and makes it impossible to resolve vibrational modes. Therefore, in Raman spectroscopy, selection of the excitation source is a crucial issue [1].

Raman and infrared absorption spectroscopies have also been widely used to probe the bond configuration of dilute nitride semiconductors [2]. The presence of N in a host III-V material restructures the conduction band of the host material, and this modification has been well explained using the interaction between the localized N level and the extended conduction band state of the host material in terms of the band anti-crossing model [4]. As a result of the interaction, the conduction band of Ga(In)As splits into two sub-bands, *E*_*−*_ and *E*_*+*_. The *E*_*−*_ band constitutes the fundamental band edge of the Ga_1−*x*_In_*x*_N_*y*_As_1−*y*_ alloy. Even a small percentage of N causes a large redshift of the band gap; therefore, incorporation of N into the III-V lattice brings more flexibility to tailor the band gap of the material [5]. However, the optical quality of dilute nitrides is drastically affected by the presence of N. Post- or *in situ* thermal annealing has been used as an effective method to improve optical and crystal qualities, but this process is responsible for a significant blueshift of the band gap energy [6,7]. The reason of the blueshift has been theoretically explained with the considerations of re-arrangement of the nearest neighbour configuration of the N environment [8] and re-shaping of quantum well (QW) from a square to a parabolic-like shape due to Ga-In interdiffusion [6]. Raman and infrared (IR) absorption spectroscopies have been used as experimental tools to confirm the theory [2].

In this work, we present the Raman scattering spectroscopy carried out with different laser lines (532, 785 and 1064 nm) in order to reveal the importance of the excitation source energy on the nature of the Raman scattering line shape as electronic, vibrational or both. Photoluminescence (PL) measurements were used to determine the electronic band gap energy of as-grown and annealed n- and p-type modulation-doped Ga_1 − *x*_In_*x*_N_*y*_As_1 − *y*_/GaAs single QW structures with *y* = 0%, 0.9%, 1.2% and 1.7%. The results obtained with the 785 and 1064 nm excitation sources showed that when the energy of the excitation source is close to the electronic band gap energy of any constituent semiconductor material in the sample, a broad peak, which was supposed to stem from the electronic transitions, dominated the spectrum, making it impossible to resolve vibrational modes of respective semiconductors. A comparison between the results of Raman and PL spectroscopies supported our interpretation of Raman scattering spectroscopy. Both techniques followed the same trend under different N concentrations and thermal annealing process. On the other hand, using Micro-Raman scattering spectroscopy with the 532-nm line, even when the excitation energy is much higher than the band gap of the GaAs or Ga_1 − *x*_In_*x*_N_*y*_As_1 − *y*_, only vibrational modes were observed. However, the intensity of the vibration modes has been observed to be very weak due to the multi-layered structure of the samples. In order to analyse the effect of the N amount and thermal annealing on vibrational modes, attenuated total reflectance (ATR) IR absorption method is preferred as a complement to the micro-Raman scattering results.

## Methods

**Figure 1:**
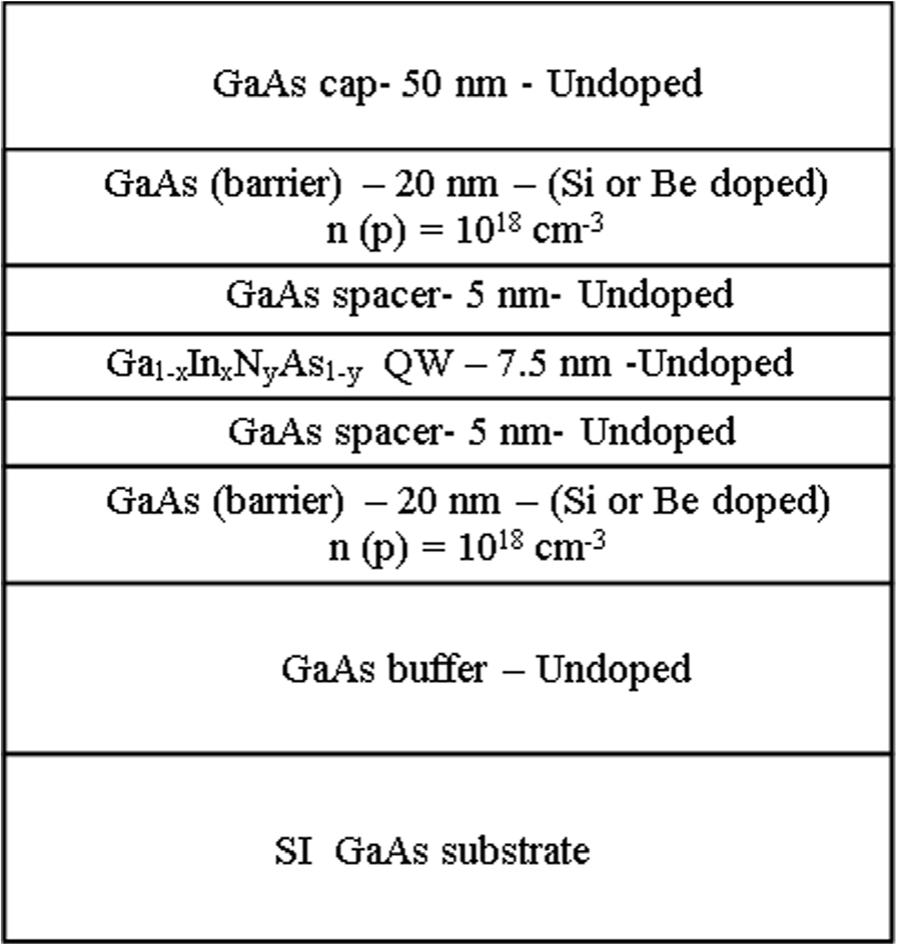
The layer structure of the studied samples.

The samples were grown on semi-insulating GaAs (100) substrates by solid-source molecular beam epitaxy (MBE) equipped with a radio frequency plasma source for nitrogen incorporation. The structures comprised 7.5 nm-thick QWs with an indium concentration of 32% and a varying nitrogen concentration. Growth temperatures for the GaAs, Ga_1−*x*_In_*x*_N_*y*_As_1−*y*_ and Ga_1 − *x*_In_*x*_As_*y*_ layers were 580°C, 475°C and 540°C, respectively. Doping levels were calibrated by Hall measurements with separate samples. Post-growth rapid thermal annealing was done at temperatures of 700°C for both 60 s and 600 s for all structures. During thermal annealing process, the samples were capped with a GaAs wafer to prevent As evaporation. The layer structures of the samples and description of the samples are given in Figure 1 and Table 1, respectively.

**Table 1:** Descriptions and the codes of the samples

**Code**	**Doping**	**Description**
TPRH	p	In_0.32_Ga_0.68_As
TPRHA		
TPRHB		
TP09	p	Ga_0.68_In_0.32_N_0.009_As_0.991_
TP09A		
TP09B		
TP12	p	Ga_0.68_In_0.32_N_0.012_As_0.988_
TP12A		
TP12B		
TP17	p	Ga_0.68_In_0.32_N_0.017_As_0.983_
TP17A		
TP17B		
TNRH	p	In_0.32_Ga_0.68_As
TNRHA		
TNRHB		
TN09	p	Ga_0.68_In_0.32_N_0.009_As_0.991_
TN09A		
TN09B		
TN12	p	Ga_0.68_In_0.32_N_0.012_As_0.988_
TN12A		
TN12B		
TN17	p	Ga_0.68_In_0.32_N_0.017_As_0.983_
TN17A		
TN17B		

Micro-Raman spectra (100 to 3200 cm^−1^) of the samples were recorded using a Jasco NRS 3100 micro-Raman spectrometer (Jasco Corporation, Tokyo, Japan), equipped with 532 nm (green) and 785 nm (red) diode lasers, whereas Fourier transform (FT)-Raman scattering measurements were carried out using a Bruker MultiRam FT-Raman spectrometer (Bruker Optik GmbH, Ettlingen, Germany), equipped with a 1064 nm Nd-YAG laser. All Raman scattering measurements were done at back-scattering geometry at room temperature. The IR spectra of the samples were recorded on a Bruker Tensor-27 FT-IR spectrometer using a diamond ATR attachment in the 4,000 to 200 cm^−1^ range with a 1 cm^−1^ resolution.

On the other hand, PL spectra were not taken with a commercial spectrometer but with a special home-designed set-up, equipped with an Ar-ion laser (514 nm) as excitation source, thermoelectric cooled GaInAs photodetector and 0.5 m monochromator. The power of the laser was kept at 70 mW.

## Results and discussions

**Figure 2:**
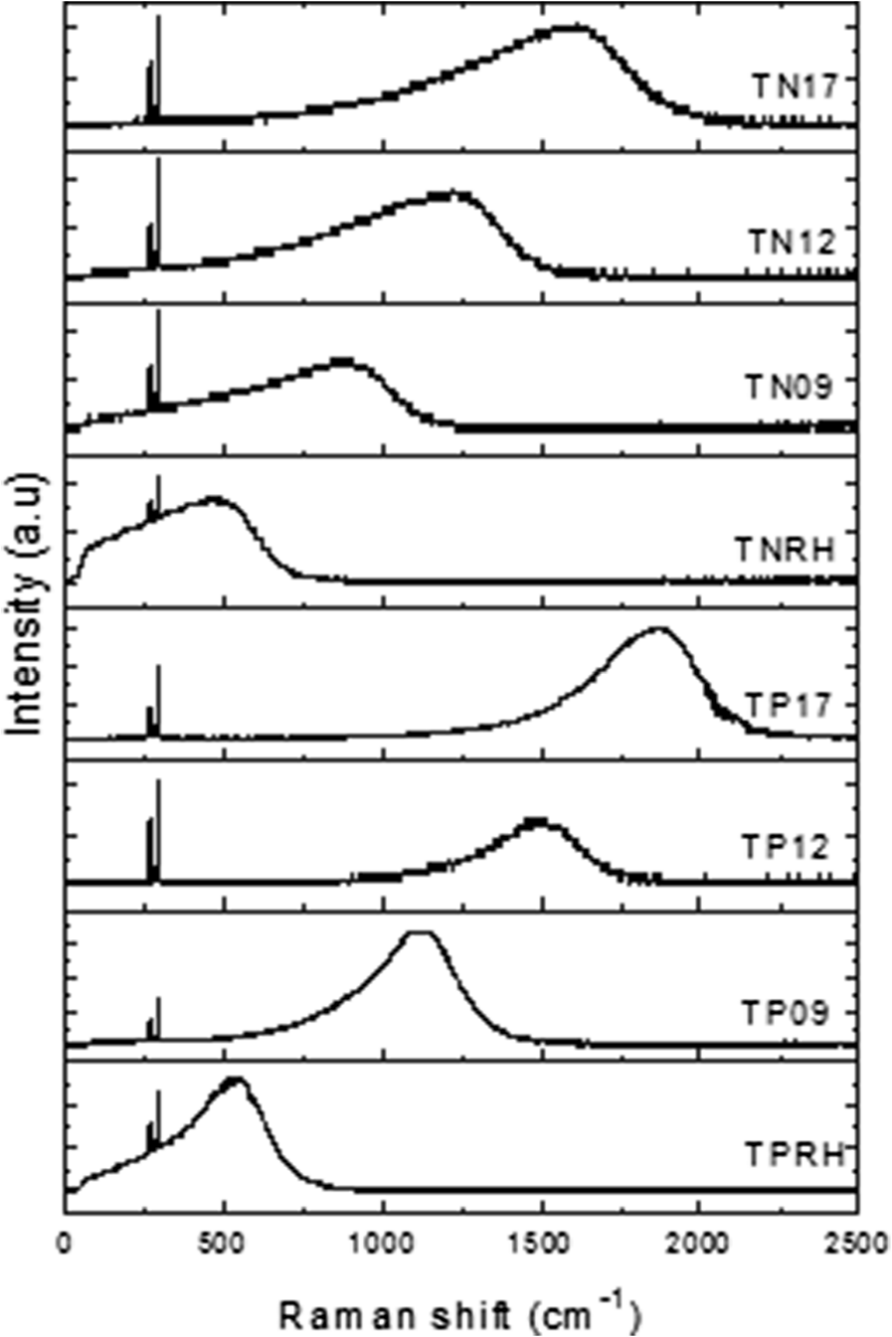
FT-Raman spectra for as-grown samples at room temperature.

Figure 2 shows the FT-Raman spectra with the 1064 nm line (1.165 eV >*E*_*g*_ (Ga_1 − *x*_In_*x*_N_*y*_As_1 − *y*_)) for as-grown samples. The FT-Raman spectrum has a very broad peak and two sharp peaks at 268 cm^−1^ (33.2 meV) and 291 cm^−1^ (36.0 meV). The wavenumber of the vibrational modes corresponds to transverse (268 cm^−1^) and longitudinal optic phonons (291 cm^−1^) and does not exhibit any change with an increasing N amount or the type of samples. Therefore, it is obvious that they are related to the phonon modes in the GaAs semiconductor in the sample. The broad peak shifts towards higher wavenumbers as N amount changes from 0% to 1.7%. In order to probe the origin of this peak, the absolute wavenumber ${\bar{\nu }}_{\text{abs}}$ is calculated using

(1) $${\bar{\nu }}_{\text{abs}}={\bar{\nu }}_{\text{laser}}-{\bar{\nu }}_{\text{measured}}=9,398.5-{\bar{\nu }}_{\text{measured}}$$

The values in energy units are tabulated in Table 2 for all samples along with PL peak energy values. PL spectra and their detailed interpretation are not given here since they are just used here to identify the origin of the broad peaks in Raman shift spectra.

**Table 2:** PL peak energies and FT-Raman absolute peak energies

**Sample code**	**PL peak energy (eV)**	**FT-Raman absolute peak energy (eV)**
TPRH	1.108	1.107
TPRHA	1.111	1.108
TPRHB	1.121	1.118
TP09	1.028	1.027
TP09A	1.055	1.057
TP09B	1.077	1.076
TP12	0.984	0.980
TP12A	1.007	1.029
TP12B	1.034	1.037
TP17	0.935	0.933
TP17A	0.969	0.975
TP17B	0.997	0.994
TN09	1.060	1.055
TN09A	1.066	1.059
TN09B	1.074	1.068
TN12	1.027	1.015
TN12A	1.032	1.022
TN12B	1.037	1.032
TN17	0.978	0.979
TN17A	0.994	0.980
TN17B	0.998	0.994

**Figure 3:**
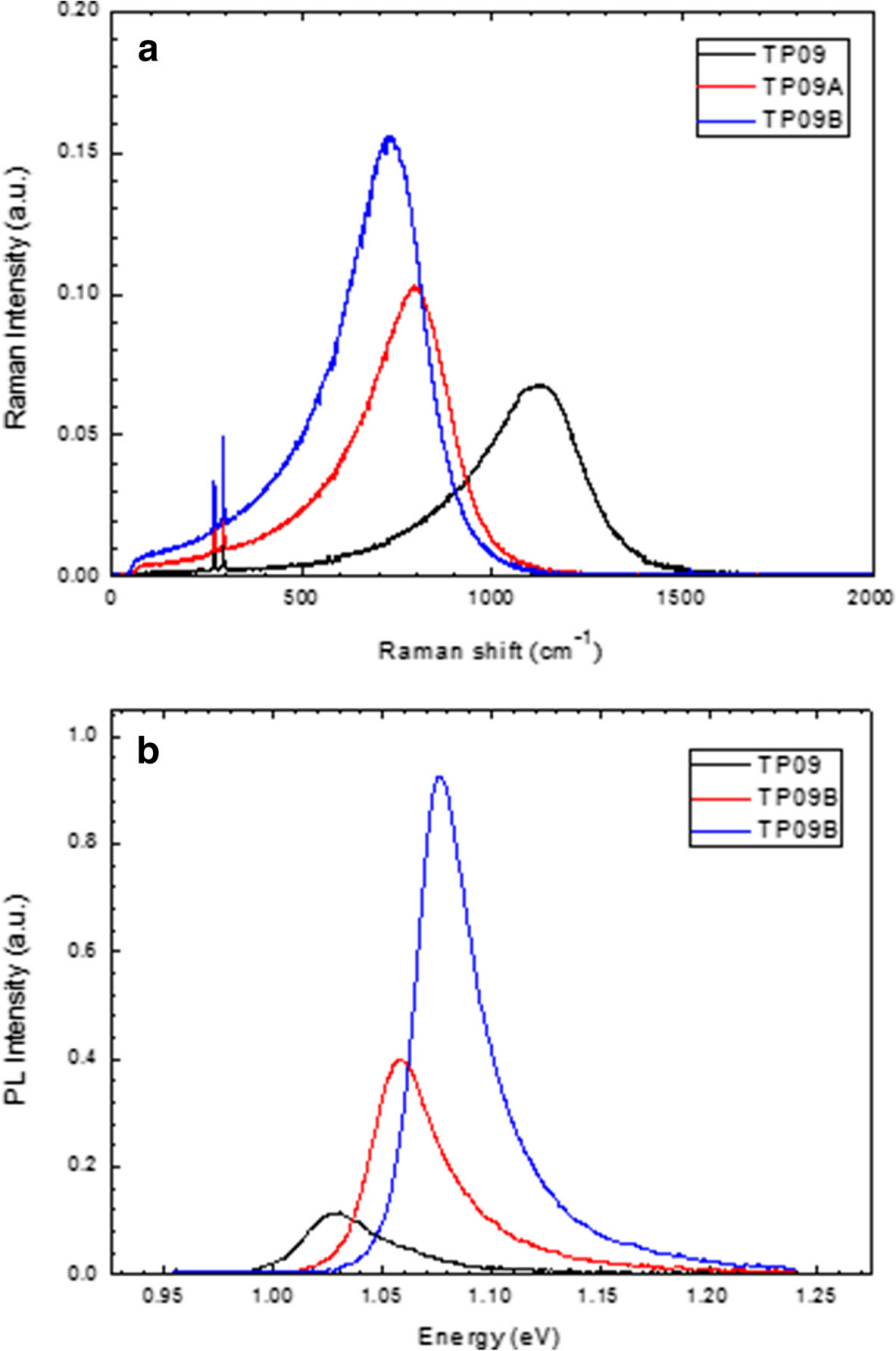
**Spectra of as-grown and thermal annealed p-type samples with N = 0.9%.** (**a**) FT-Raman and (**b**) PL spectra.

In Figure 3, effects of thermal annealing have been investigated using FT-Raman scattering and PL results for the p-type sample containing 0.9% N. It is well known that thermal annealing improves both optical and crystal qualities but causes a significant blueshift of the band gap in Ga_*x*_In_1 − *x*_N_*y*_As_1 − *y*_ alloys. In terms of thermal annealing, we observed that the electronic band structure of the samples in both Raman scattering and PL measurements was affected in the same manner.

As can be seen in Table 2, there is a good match between FT-Raman scattering (using the absolute value of the wavenumber) and PL results. Therefore, it is obvious that the broad peaks observed in the Raman shift spectra originate from the recombination of electron and hole emitting a photon whose energy corresponds to the effective band gap of the Ga_*x*_In_1−*x*_N_*y*_As_1−*y*_ semiconductor in the sample. Because the luminescence is so strong, it suppresses the possible vibrational modes related to the presence of the N atom in the host lattice which are expected to be observed between 400 and 600 cm^−1^[9].

**Figure 4:**
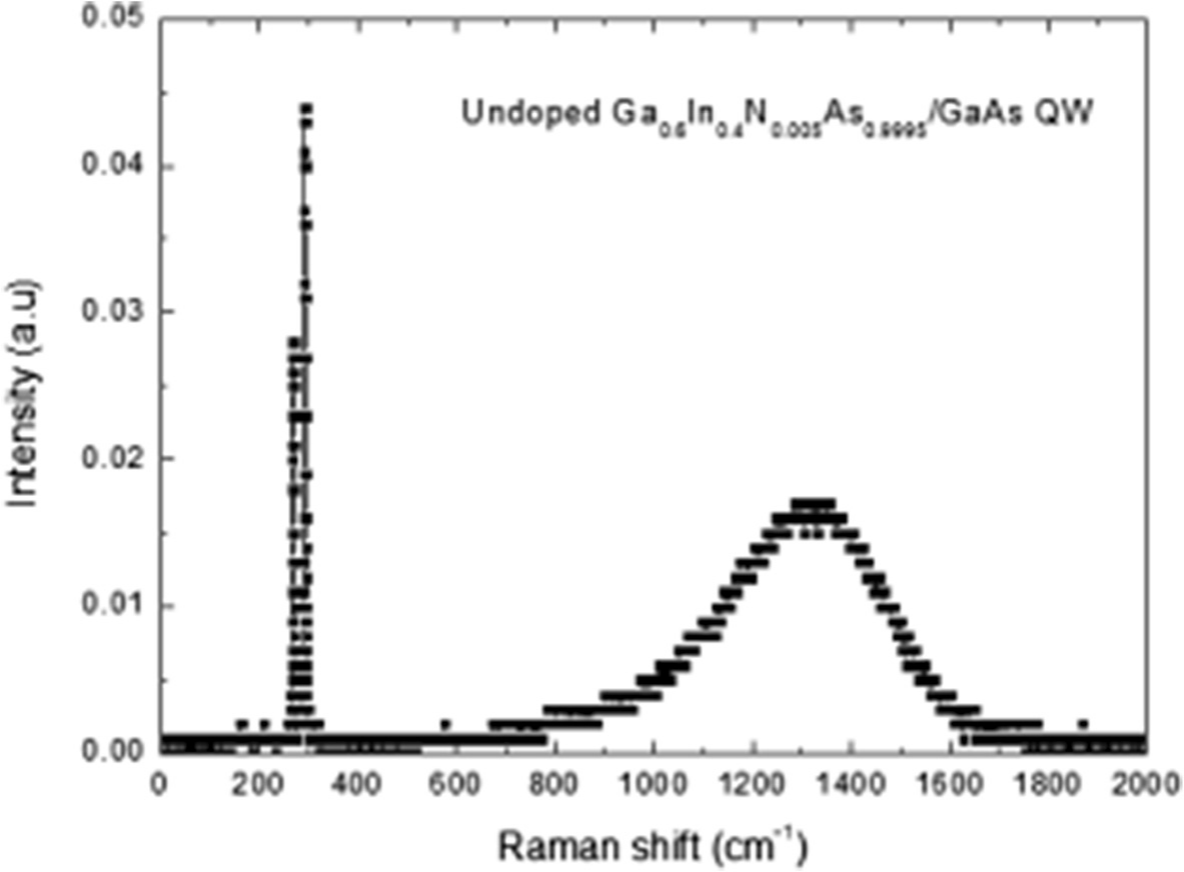
**FT-Raman spectrum of undoped Ga**_**0.60**_**In**_**0.40**_**N**_**0.005**_**As**_**0.995**_**/GaAs QW structure.**

Since the samples are doped, considering that all dopant atoms are ionized at room temperature, the existence of the broad peak feature in Raman scattering spectra may be interpreted as LO-optical phonon coupled modes. We have also taken into account this possibility to identify the mechanism behind the observed broad peaks. Therefore, FT-Raman scattering measurements have been carried out an undoped Ga_*x*_In_1−*x*_N_*y*_As_1−*y*_/GaAs QW sample. As seen in Figure 4, again a broad peak was observed. When the peak energy calculated from Equation 1 is compared to the PL peak energy, we obtained a good match [10]. This result also confirms that the observed broad peak did not originate from LO-plasmon coupled modes but from the electronic transition in the Ga_*x*_In_1 − *x*_N_*y*_As_1 − *y*_/GaAs QW.

**Figure 5:**
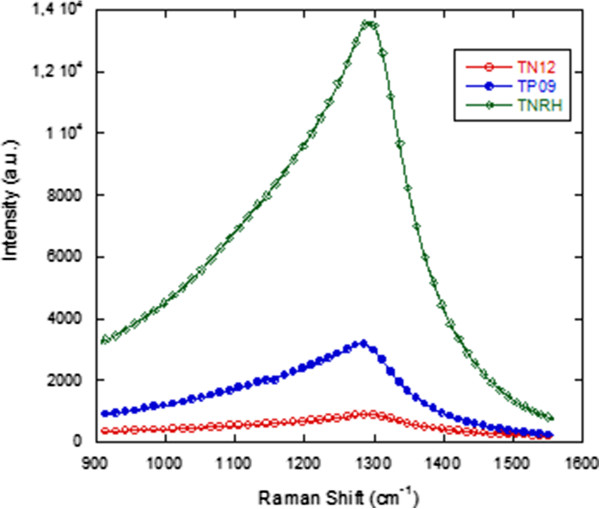
Micro-Raman spectra with 785-nm laser.

**Figure 6:**
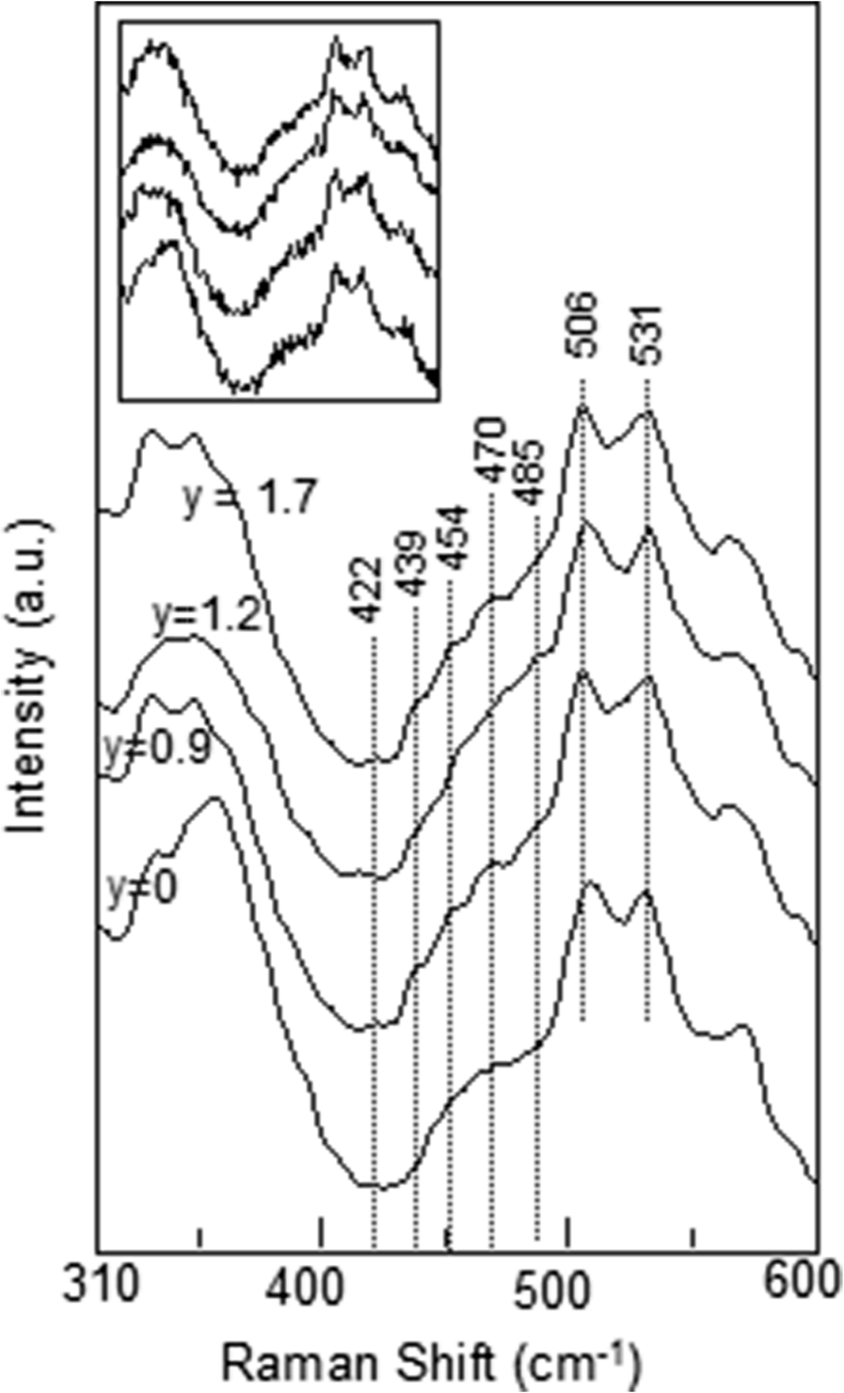
**Micro-Raman spectra with 532-nm laser of n-type Ga**_**0.68**_**In**_**0.32**_**N**_***y***_**As**_**1 − *y***_**/GaAs QW samples with different N concentrations.** The inset shows the unsmoothed raw Raman spectra of the samples

**Figure 7:**
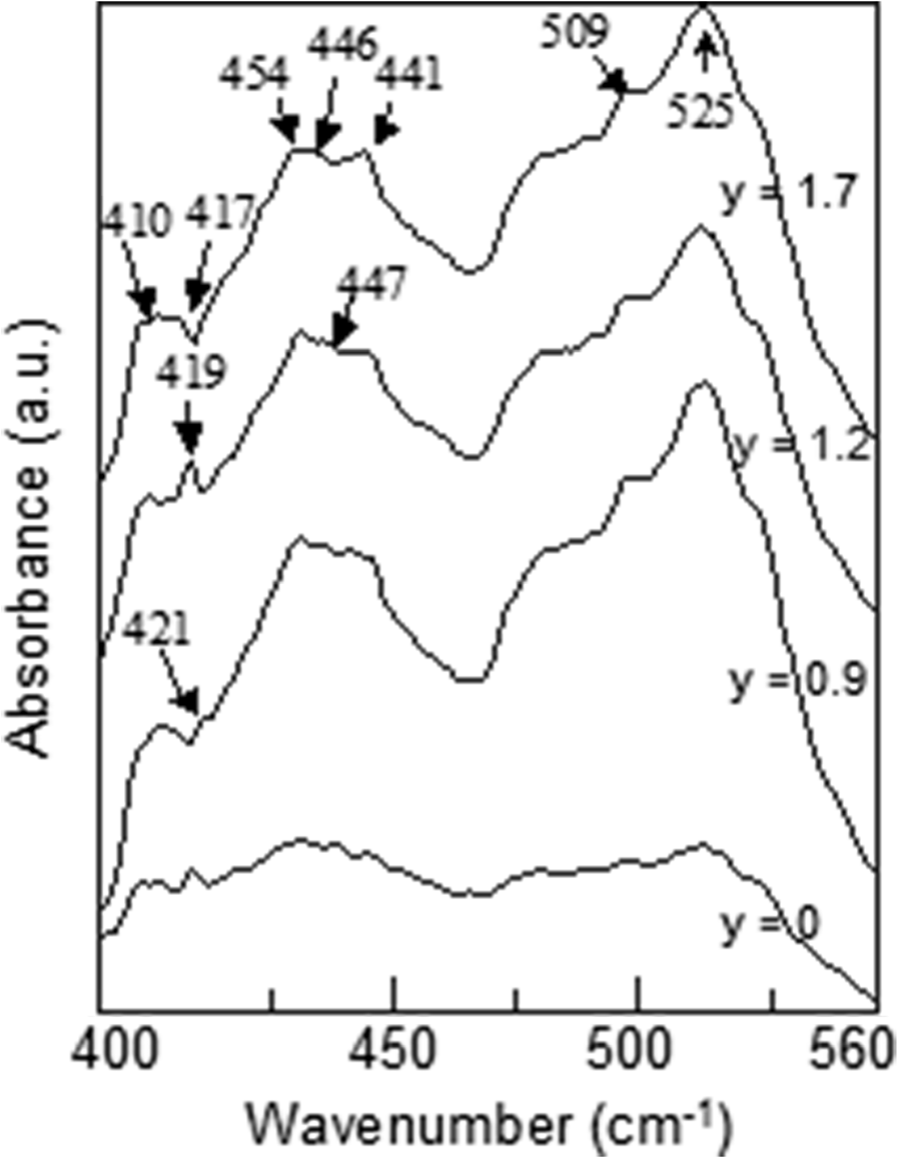
**ATR IR spectra of n-type Ga**_**0.68**_**In**_**0.32**_**N**_***y***_**As**_**1 −** ***y***_**/GaAs QW samples with different N concentrations.**

**Figure 8:**
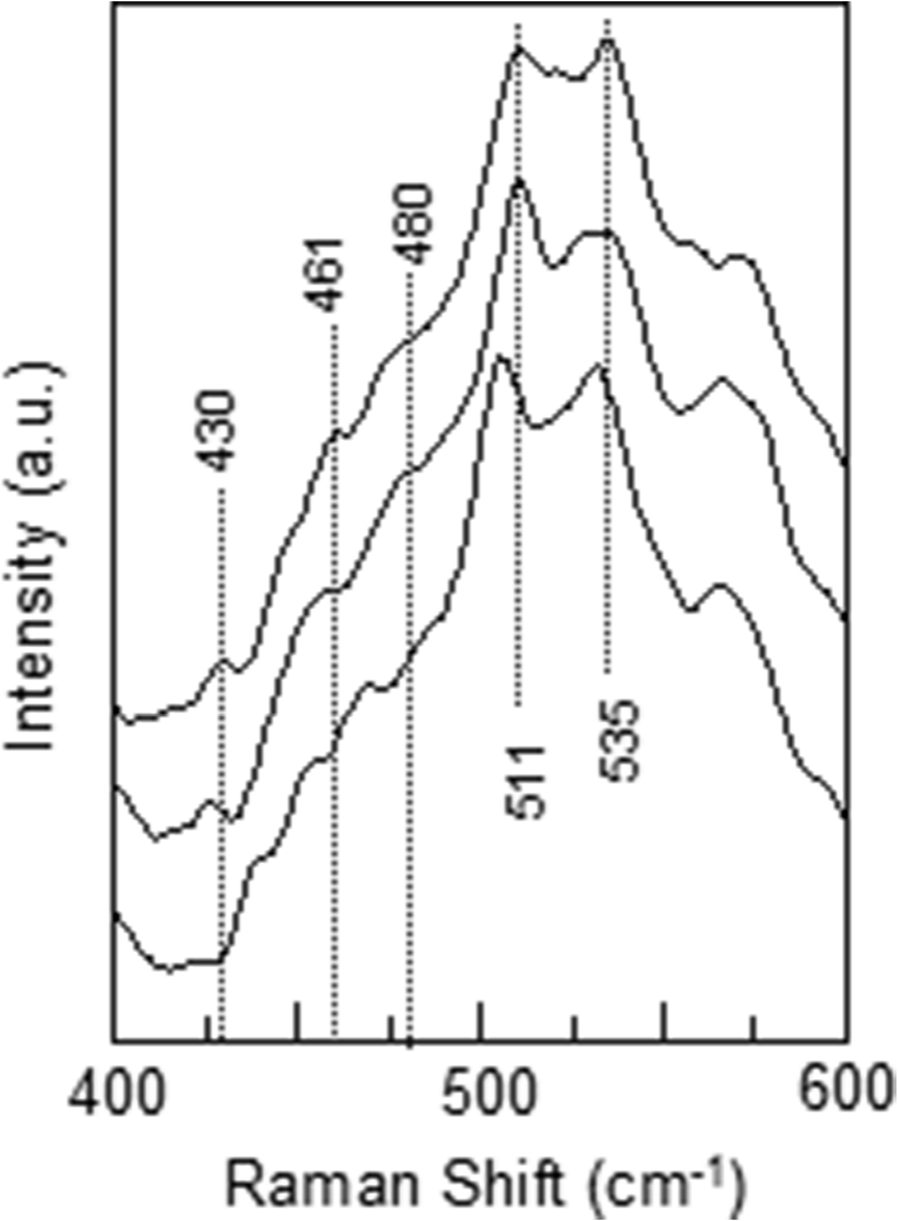
Raman spectra of TN17, TN17A and TN17B.

**Figure 9:**
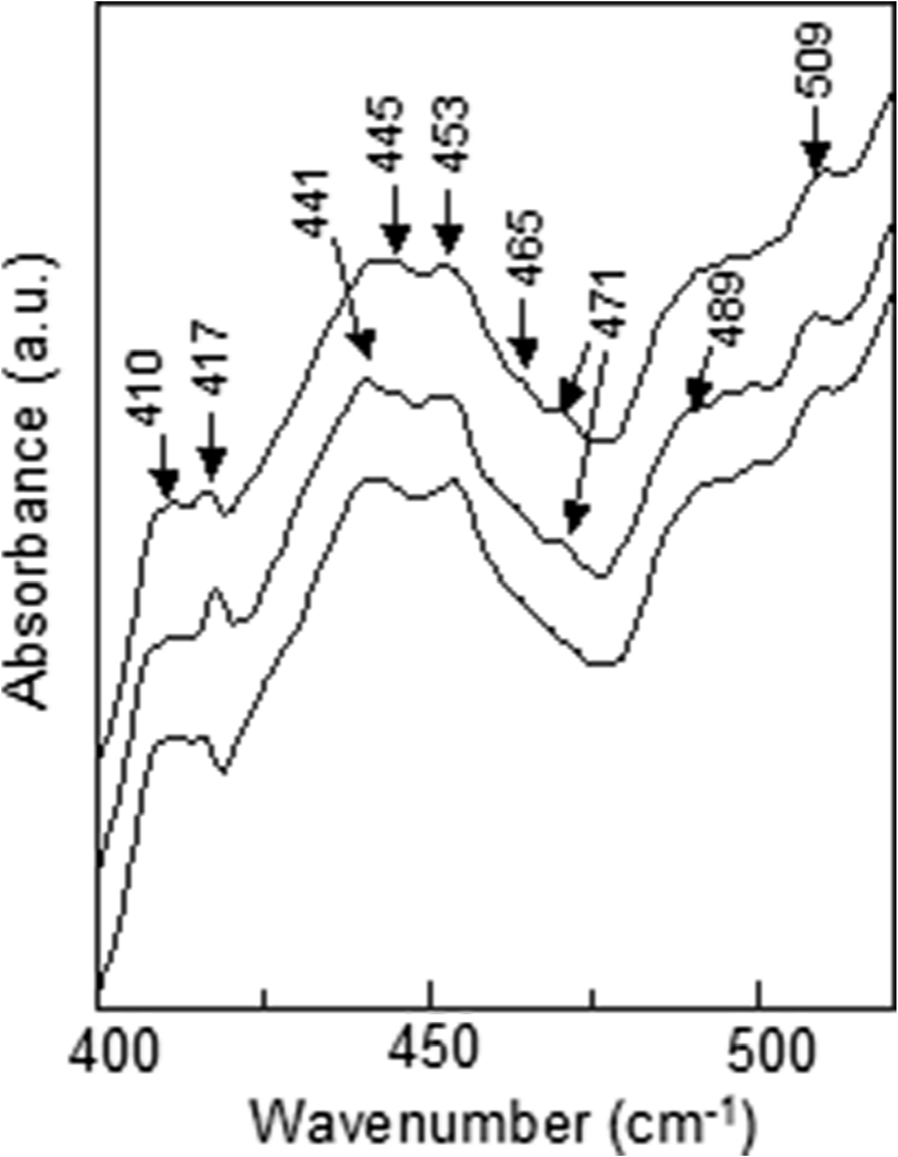
ATR IR spectra of TN17B (top curve), TN17A (middle curve) and TN17 (bottom curve).

Micro-Raman scattering measurements with the 785-nm line (*E*_laser_ = 1.59 eV, ${\bar{\nu }}_{\text{laser}}$ = 12738.8 cm^−1^) of a diode laser have also exhibited a broad band feature (see Figure 5 for three of the samples). We found that the absolute energy of the broad peak corresponds to the band gap of GaAs layer in the samples. In the light of the aforementioned findings and interpretations, it can be predicted that unless excitation energy is much higher than the electronic transition of constituent semiconductors, GaAs and Ga_*x*_In_1 − *x*_N_*y*_As_1 − *y*_, in the sample, it is not possible to observe vibrational modes. The vibrational characteristic at Raman scattering spectra of the samples has been only observed by micro-Raman spectroscopy with the 532-nm (2.33 eV >>*E*_g_ (GaAs) and *E*_g_ (Ga_1 − *x*_In_*x*_N_*y*_As_1 − *y*_)) laser line. Since the excitation energy is much higher than the band gap energies (approximately 1 eV) of GaAs and/or Ga_1 − *x*_In_*x*_N_*y*_As_1 − *y*_, electrons are excited to much higher levels in the conduction band, and the excess energy of the electrons is spent via multiple phonon scatterings before radiative band-to-band transition occurs. The more excess energy enables more electron–phonon scatterings, and that makes the vibrational modes appear in Raman scattering spectra. Figures 6, 7, 8 and 9 show the results of micro-Raman scattering along with ATR IR absorption. Normally, the intensity of inelastic scattering is quite small, and it is difficult to observe and analyse vibrational modes. Therefore, IR absorption technique is more suitable for the local vibrational mode (LVM) analysis. However, since the samples are multi-layered, we could not observe any transmitted light from the backside of the sample and used ATR IR absorption technique to be able to complement the micro-Raman results. Although the analysis of the vibration spectroscopy is beyond the scope of the paper, we give a brief discussion here, leaving the detailed analysis for a future work.

LVM analysis of Ga_1 − *x*_In_*x*_N_*y*_As_1 − *y*_ alloys has been studied by many authors [9,11-17] to investigate the local environment of N. In one of these studies [11], the LVM of N-implanted In_*y*_Ga_1 − *y*_As grown by MBE method has been reported, and it was shown that with increasing In content in the epitaxial layer, the formation of In-rich environments was more pronounced than is expected for a random distribution of the constituents. The authors have observed two N-related LVMs at 447 and 510 cm^−1^ in a sample with an In content of more than 20% (In_0.26_Ga_0.74_As). They proposed N-GaIn_3_ configuration for these bands with C_3v_ symmetry. As can be seen in Figure 7, we also observed the same peaks at *ca.* 447 and 509 cm^−1^.

When N concentration is increased, the peak at *ca.* 420 cm^−1^ shifts down to 417 cm^−1^, and the relative intensity of the peak at approximately 410 cm^−1^ decreases. Peaks around 409 and 426 cm^−1^ have been reported before as related to N dimers [12,13], but in this study, the peak at 410 cm^−1^ also appear in Raman spectrum for the reference sample (*y* = 0%). It is probably either In-related mode or Si-related mode since the GaAs barriers used are Si-doped, and the Si mode of Si-doped GaAs is known to be at approximately 399 cm^−1^. The peak is further affected by annealing process (see Figure 9).

It is also worth mentioning that a peak at approximately 430 cm^−1^ is slightly forming during the annealing process (see Figure 8). A peak at 434 cm^−1^ in the Raman spectrum of the GaInNAs quantum well has been observed after annealing [12], and it was proposed to be related to an In and NN_As_ dimer complex as can be the case in our sample. Also, the annealing procedure reveals the well-known 471 cm^−1^ Ga-N peak slightly (see Figure 9).

We report here the raw data obtained from ATR IR and Raman spectra on n-type modulation-doped Ga_0.68_In_0.32_N_y_As_1−*y*._ To assign each LVM mode correctly, further analysis of the spectra is needed like subtracting the reference spectra and band component analysis and/or second derivative analysis of the corresponding spectra. Also, the LVM analysis of p-type samples is left for a future study.

## Conclusions

We have investigated the excitation energy-oriented nature of the Raman scattering spectrum using FT-Raman and micro-Raman techniques with different excitation sources on as-grown and annealed n- and p-type Ga_1 − *x*_In_*x*_N_*y*_As_1 − *y*_/GaAs QW samples. ATR IR absorption and PL measurements were used to complement the Raman scattering results. FT-Raman scattering results exhibited a broad peak and two sharp peaks. A comparison between PL and FT-Raman absolute values revealed that the broad peak corresponds to the electronic transition in the Ga_1 − *x*_In_*x*_N_*y*_As_1 − *y*_/GaAs QW and gives the well-known effect of N and thermal annealing on the band structure of the host material, i.e*.* In_*x*_Ga_1 − *x*_As. On the other hand, micro-Raman with the 532 nm line of excitation source, whose energy is much higher than the electronic band gap energy of both GaAs and Ga_1 − *x*_In_*x*_N_*y*_As_1 − *y*_, exhibited only vibrational modes. The results indicate that Raman spectroscopy can provide useful information for electronic and/or vibrational modes depending on the energy of excitation source. The effect of N and thermal annealing on chemical bond configuration is investigated using micro-Raman (with 532 nm excitation) and ATR IR absorption. We have proposed that the N environment in the samples changed as post-growth annealing was applied, and to clearly understand this new environment, further analysis of the experimental data should be made.

## Abbreviations

ATR: Attenuated total reflectance; FT: Fourier transform; IR: Infrared; LVM: Local vibrational modes; MBE: Molecular beam epitaxy; PL: Photoluminescence; QW: Quantum well.

## Competing interests

The authors declare that they have no competing interests.

## Authors’ contributions

AE designed the samples, wrote the article and supervised the PL experiments. EA wrote the local vibrational analysis part of the article and carried out the FT-Raman and micro-Raman experiments. FS and OD carried out the PL experiments and helped analyse the results. SA carried out the IR ATR experiments. MCA supervised the PL experiments. JP and MG grew and annealed the designed samples. All authors read and approved the final manuscript.
